# How functional genomics will impact fruit fly pest control: the example of the Mediterranean fruit fly, *Ceratitis capitata*

**DOI:** 10.1186/1471-2156-15-S2-S11

**Published:** 2014-12-01

**Authors:** Francesca Scolari, Ludvik M Gomulski, Paolo Gabrieli, Mosè Manni, Grazia Savini, Giuliano Gasperi, Anna R Malacrida

**Affiliations:** 1 Department of Biology and Biotechnology, University of Pavia, Pavia, Italy

**Keywords:** Medfly, Sterile Insect Technique (SIT), functional genomics, transcriptomics, tephritids

## Abstract

The highly invasive agricultural insect pest *Ceratitis capitata *(Diptera: Tephritidae) is the most thoroughly studied tephritid fruit fly at the genetic and molecular levels. It has become a model for the analysis of fruit fly invasions and for the development of area-wide integrated pest management (AW-IPM) programmes based on the environmentally-friendly Sterile Insect Technique (SIT). Extensive transcriptome resources and the recently released genome sequence are making it possible to unravel several aspects of the medfly reproductive biology and behaviour, opening new opportunities for comparative genomics and barcoding for species identification. New genes, promotors and regulatory sequences are becoming available for the development/improvement of highly competitive sexing strains, for the monitoring of sterile males released in the field and for determining the mating status of wild females. The tools developed in this species have been transferred to other tephritids that are also the subject of SIT programmes.

## Background

The Mediterranean fruit fly (medfly), *Ceratitis capitata *Wiedemann, is one of the world's most destructive agricultural insect pests [1-3]. Due to its global distribution and history of rapid and devastating outbreaks [4-6], the medfly is the most thoroughly studied "true" fruit fly (Diptera: Tephritidae) [7] at the genetic and molecular levels. It has thus become a model species for the analysis of fruit fly invasions [8] and for the development of control strategies [9]. Medfly outbreaks have been successfully controlled through area-wide integrated pest management (AW-IPM) programmes based on the environmentally-friendly Sterile Insect Technique (SIT) [10]. In the SIT, the reduction of pest population size is achieved through mass release of reproductively sterile male insects into a wild-type population [11]. Males rendered sterile through ionizing radiation compete with wild-type males for matings and deplete female reproductive success. Preventative sterile male releases have been and are currently applied in areas where the climatic conditions and the availability of suitable hosts for oviposition are particularly favourable for medfly establishment, such as California, Southern Australia and Florida [12-16]. To be most successful, this approach requires i) knowledge of the genetic background of the released males and the genetic structure of the target population, ii) a sexing strain for male-only production, iii) a sterilization system that inflicts the least possible fitness load, and iv) effective procedures to monitor the efficiency of the programmes.

**Figure 1:**
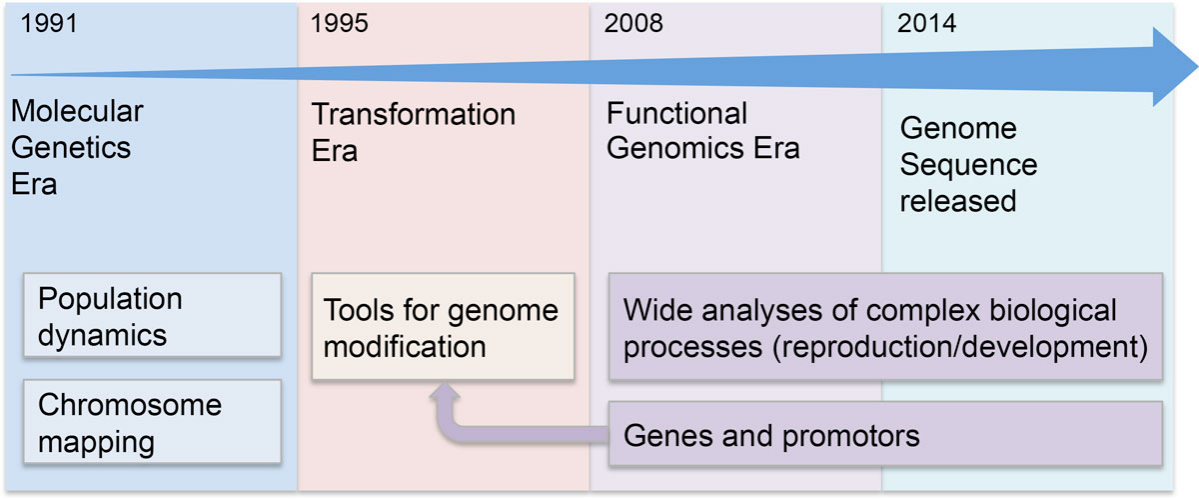
**Molecular timeline of medfly research**.

In the last 20 years, enormous progress has been made in understanding medfly biology, with the goal of developing and optimizing a wide range of molecular tools for the implementation of population control strategies (Figure 1). Population genetics provided useful approaches for reconstructing the routes of medfly invasion, highlighting the complexity of the process [4,5,17-25]. *Ceratitis capitata *was the first non-drosophilid species in which the germ-line was transformed [26], enabling studies on its biology in ways that were previously impossible [27-34].

**Figure 2:**
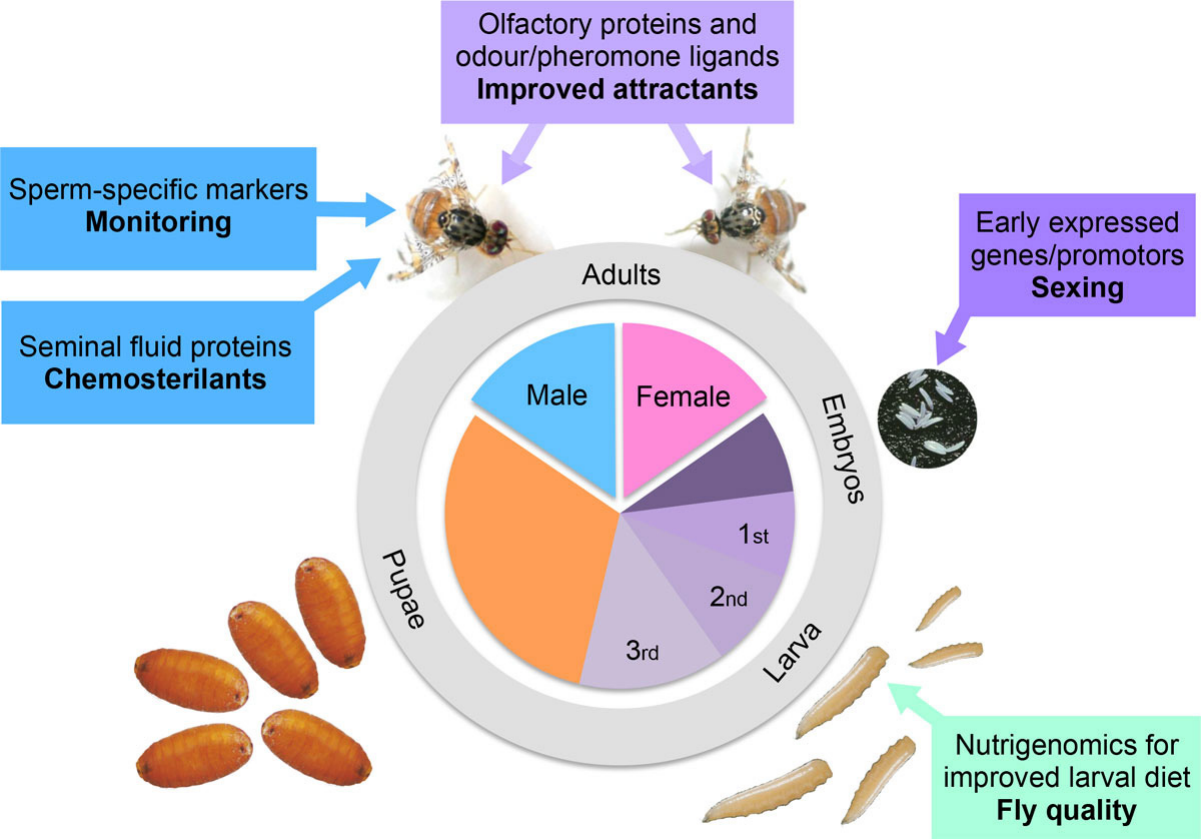
**Medfly functional genomics resources and their impact on the improvement of the SIT**.

The application of functional genomics tools, together with the recent release of the medfly genome sequence (http://arthropodgenomes.org/wiki/i5K;https://www.hgsc.bcm.edu/arthropods/medfly-genome-annotation-groups), allows a more detailed analysis of the complex biological traits that underpin the adaptive potential of this fly at all developmental stages (Figure 2)[8,35]. Indeed, functional genomics provides powerful evolutionary tools to interpret how medfly (either wild or transgenic) develop and respond to the environment. Different aspects of development, behaviour, sexual maturation, and reproduction can now be examined both in terms of gene expression profiles and protein analyses [36-43]. New genes, promotors and regulatory sequences are consequently becoming available for i) the development/improvement of competitive sexing strains, ii) the monitoring of released males in the field, and iii) for determining the mating status of wild females.

## Medfly embryogenesis

A reservoir of early male/female differentially expressed genes and sex regulatory sequences is now available for unravelling the first steps of medfly embryogenesis, i.e. when the maternal-to-zygotic transition (MTZ) occurs and when the sexual fate is established at the molecular level [36,38]. As a practical consequence, promotor and enhancer sequences that are active in early stages of development are becoming available as tools for the future generation and/or improvement of the existing conditional embryonic and female-specific lethality systems developed using conventional techniques. Female-specific lethality systems were developed based on alternative splicing of the *Cctransformer *gene (*Cctra*) [31]. Moreover, cellularisation-specific promotors/enhancers allowed the development of a transgenic embryo-specific lethality system [33]. More recently, the combination of the *Cctra*-based female-specific lethality [31] with the embryonic lethality system [33], yielded a female-specific embryonic lethality (FSEL) system in this species [44].

In this context, the medfly genes with vital functions in early embryonic development, such as those involved in sex determination and cellular blastoderm formation, are of direct use [38]. Their zygotic transcriptional activation follows two waves. The first wave starts within four hours after oviposition and includes the zygotic genes *Ccsisterless A *(*CcsisA*), *Ccdeadpan *(*Ccdpn*) and *Ccslow-as-molasses *(*Ccslam*). The second major burst of expression activation begins five hours after oviposition and includes the maternal-zygotic genes *Ccgroucho *(*Ccgro*), *CcSex-lethal *(*CcSxl*), *Cctransformer *(*Cctra*), *Ccfemale-lethal d *(*Ccfl(2)d*), *CcRho1 *and *Ccserendipity-α *(*Ccsry-α*)[38]. During this transition, sexual identity is established at the molecular level, before cellularisation of the embryo occurs. Unlike *Drosophila *[46,47], *Cctra *is the key-gene of the sex-determination cascade: it generates mRNAs encoding full-length active proteins only in females and displays an autocatalytic function, that guarantees the female-specific development of cell memory [47]. *Cctra*, in cooperation with *Cctra2*, determines the sex-specific splicing of *Ccdsx*, the transcription factor that is the regulator of the sex-differentiation processes. The mother supplies the embryos with *Cctra *and *Ccdsx *female-specific splicing variants. Subsequently, the maternal information for female-specific development is reset in embryos through the reprogramming of *Cctra *mRNA splicing and the degradation of the maternal *Ccdsx *mRNAs [38]. The precise timing of sex-specific splicing [38], as well as the proof of evidence that transgenic dsRNA for *tra *is effective in the conditional production of male-only progeny [48], can be exploited for the development of novel sexing strains.

## Metabolic regulation of sexual maturation and mating

The production of highly competitive males is an essential requisite for effective SIT. Transcriptome and microarray-based functional analyses performed on whole flies and specific tissues are providing basic information on the pathways involved in primary metabolism, hormone synthesis, neurological-related processes, gametogenesis, signalling, and sensory perception [36,37,39,42]. The regulation of these specific pathways and biological processes can be affected by long-term artificial rearing, that may translate into reduced quality of individuals released for SIT [42]. In particular, down-regulation of signaling and neurological processes, especially those related to light and chemical stimuli, muscle development, muscle differentiation and locomotion, have been reported as a consequence of mass rearing in artificial conditions [42]. In this context, nutrigenomics can provide valuable information on how nutrition affects gene expression patterns, offering the means to measure male and female medfly responses to changes in the food stream, but also providing information on diet limitations [49]. This is a priority for operational SIT. Transcriptional baseline profiles of key biological pathways involved in sexual maturation of both sexes, and also in response to mating, are available for medflies reared on the standard diet used in mass rearing facilities [37]. Indeed, we know that medfly female maturation requires the activation of fatty acid metabolism as a reflection of the high energy requirements for female reproductive success, such as foraging, nutrient storage and egg development [50]. In addition, Gene Ontology (GO) enrichment analyses revealed that, in mature females, transcript categories related to memory/learning behaviours and visual and olfactory functions are significantly overrepresented [37]. By contrast, male sexual maturation requires the activation of carbohydrate and protein metabolism for energy production and muscle activities, memory formation, smell recognition and pheromone production [37]. All these activities suggest an investment required for lek formation and courtship [51]. Despite extensive post-mating transcriptional changes in the male, changes in the female were surprisingly modest. Indeed, in the male, mating does not down-regulate the transcriptional activities of genes implicated in lek formation/courtship, whereas it increases the activities of genes related to fitness (i.e. *double time *and *Basigin*) [37].

Some of these pathways are down-regulated by irradiation [42]. This is the case of processes related to visual and chemical responses, and those associated with muscle development and locomotion. These irradiation-related changes may have an impact on the competitiveness of mass reared flies.

Studies on improved diets or chemical manipulation of the adult environment offer promising options for the improvement of sterile male competitiveness. Approaches aimed at the improvement of the sexual performance of sterile males include i) altering the olfactory environment experienced by freshly eclosed individuals, providing high-quality post-teneral nutrition [52] and ii) inoculating males with probiotic bacteria [53,54].

## Male reproduction

A better understanding of the reproductive biology of the medfly should permit the development of novel or improved approaches to impact male reproductive success and/or regulate female mating behaviour and fertility. In this respect, transcriptomics and proteomics of reproductive tissues will help to identify genes and promotors. Testes and male accessory glands (MAGs) participate in the maintenance of complementary reproductive functions. In the testes, the key regulatory genes of spermatogenesis tend to be conserved to guarantee the male-specific processes required for sperm production [55,56]. By contrast, the accessory gland secretions act as key factors in male insect reproductive success, and the genes expressed in the MAGs are subject to rapid evolution as a result of sexual conflict and competition [57]. A transcriptome-based analysis performed on medfly testes and male accessory gland tissues resulted in a database of 3344 unique sequences [39]. Transcripts related to spermatogenesis, fertility, sperm-egg binding, as well as those involved in the production of seminal fluid proteins (SFPs), were identified. Some of the SFP transcripts displayed a mating-responsive profile [39]. These will be ideal targets for the development of novel and more specific environmentally-friendly chemosterilants [58,59] that mimic the behaviour-modulating effects of MAG proteins, i.e. by impeding correct sperm storage, or interfering with female remating.

Over a third of the transcripts from these two tissues shared no significant similarities to known genes from other organisms. Considering that they may represent novel and/or fast-evolving sequences, they represent ideal targets for the development of species-specific diagnostic markers.

## Improved SIT monitoring strategies

**Figure 3:**
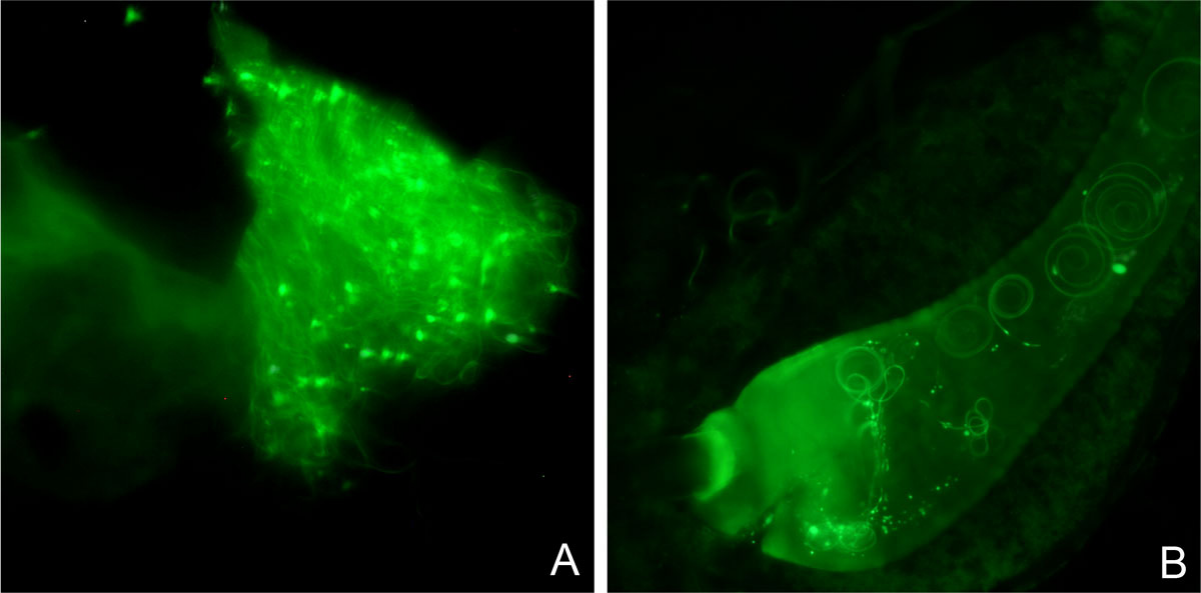
**Transgenic sperm can be easily traced in the reproductive tract of laboratory wild-type females**. Mechanically opened spermatheca isolated from a laboratory wild-type female mated with a transgenic male with green fluorescent sperm [32], three days after death (A). Spermathecal duct dissected from a laboratory wild-type female mated to a transgenic male with green fluorescent sperm [32] 24 hours after mating (B). Images were captured using an epifluorescence Zeiss Axioplan microscope at 400x magnification with the Zeiss filters set 13.

A major issue in the monitoring activities for evaluating SIT effectiveness is the difficulty in assessing the capacity of released sterile males to inseminate wild-type females [60-62]. The availability of the testes- and sperm-specific Cc*β2-tubulin *gene has allowed the use of its promotor for fluorescent protein marking of the spermatozoa, and hence to detect females that have mated with released males [32]. Using this marking system, strains have been generated and evaluated for their ability to transfer green or red fluorescent sperm to the female spermathecae. It has been proven that these sperm remain viable and fluorescent for a long time within the spermathecae, also after female death [32] (Figure 3). The transgene previously inserted in one of these lines, namely 1260_F-3_m-1, was then efficiently modified by the use of the site-specific integration system from phage *phiC31 *[34]. Post-integrational excision of one of the *piggyBac *inverted terminal repeats resulted in stably integrated transgene insertions that, being inert to the *piggyBac *transposase, could not be remobilized. This allowed the development of an optimized strain for pest control that minimizes environmental concerns (stab_1260_F-3_m-1)[34]. Once integrated into the medfly GSS Vienna-8 strain, this sperm marking system may offer valuable alternatives to the currently used fluorescent powders [63] that are detected in trapped flies using UV light. Moreover, this sperm marking system can also be integrated into strains carrying diverse transgenes in tandem, for example with conditional embryonic lethality [33] and sexing systems [31].

For monitoring activities, one of the priorities is the development of powerful species- and sex-specific attractants. In this context, it is essential to identify the components of the molecular machinery that recognizes and binds external ligands (odours and pheromone components) and translates this interaction into electrical signals to the central nervous system. Three main groups of molecules are involved: odorant-binding proteins (OBPs), chemosensory proteins (CSPs), and the chemoreceptor superfamily formed by the olfactory (OR), gustatory (GR) and ionotropic (IR) receptor families [64,65]. The chemosensory gene repertoire of the medfly is currently being characterized at the functional genomics and structural level [40,41]. So far, one antennal-enriched OBP appears to be particularly promising for practical applications. Indeed, it displayed highest binding specificity for (E,E)-α-farnesene, a major component of male pheromone blend, and also for Trimedlure, a strong synthetic medfly attractant [41]. The resolution of the three-dimensional structure of this medfly OBP will be the premise for the design of synthetic molecules able to act as antagonists of the natural ligand/s. Such optimized molecules need to be further evaluated and tested for side-effects before they can be used in AW-IPM approaches.

## Conclusions

The extensive transcriptome resources now available for the medfly (Table 1) will greatly improve the on-going annotation of the genome. They will also facilitate the generation of genomic data from other tephritid species of agricultural importance [66-71], opening new ways for comparative genomics and barcoding for species identification. In addition, the structural and functional genomics (transcriptomics, proteomics, RNA interference etc) tools that are being developed in the medfly can be extended to other tephritids that are also the subject of SIT programmes, such as *Anastrepha *and *Bactrocera *species (*A. ludens, A. suspensa, A. obliqua, A. fraterculus, B. cucurbitae, B. tryoni, B. dorsalis, B. correcta*)[10].

**Table 1:** Transcriptome and microarray resources available for the medfly.

Database	Tissue/status	Strain	Type	Accession numbers	**Ref**.
NCBI dbEST database	Embryo (from 30 min to 36 hr after oviposition)	Ispra	Expressed sequence tags	FG068301-FG078567	[36]

NCBI dbEST database	Adult male and female heads (from 30 min to 8 days after emergence).	Ispra	Expressed sequence tags	FG078568-FG089553	[36]

NCBI dbEST database	Adult testes and male accessory glands.	Ispra	Expressed sequence tags	JK832450-JK838363	[39]

NCBI dbEST database	Adult male accessory glands.	Guatemala mass-rearing strain (Moscamed)	Expressed sequence tags	DQ406805-DQ406817	[72]

NCBI GEO Dataset	Adult female head. Immature versus mature.	Ispra	Microarray	GSE19571	[37]

NCBI GEO Dataset	Adult male head. Immature versus mature.	Ispra	Microarray	GSE19572	[37]

NCBI GEO Dataset	Adult female head. Mated versus Virgin.	Ispra	Microarray	GSE19573	[37]

NCBI GEO Dataset	Adult Male head. Mated versus Virgin.	Ispra	Microarray	GSE19608	[37]

NCBI Sequence Read Archive	Adult, whole body, Irradiated	Vienna 7	Illumina Hiseq 2000 sequencing	SRX312172-SRX312174	[42]

NCBI Sequence Read Archive	Adult, whole body, Non-irradiated	Vienna 7	Illumina Hiseq 2000 sequencing	SRX312183-SRX312185	[42]

NCBI Sequence Read Archive	Pupae, Irradiated	Vienna 7	Illumina Hiseq 2000 sequencing	SRX312176-SRX312180	[42]

NCBI Sequence Read Archive	Pupae, Non-irradiated	Vienna 7	Illumina Hiseq 2000 sequencing	SRX312186-SRX312188	[42]

NCBI Sequence Read Archive	Adult, whole body, Non-irradiated	Wild Hawaii	Illumina Hiseq 2000 sequencing	SRX312189-SRX312191	[42]

NCBI Sequence Read Archive	Pupae, Non-irradiated	Wild Hawaii	Illumina Hiseq 2000 sequencing	SRX312192-SRX312194	[42]

The increased knowledge of the biology of the medfly acquired through genomic approaches will also facilitate the further development of regulations for the transfer and potential field release of genetically modified fruit flies.

## Competing interests

The authors declare that they have no competing interests.
